# Uterine macrophages as treatment targets for therapy of premature rupture of membranes by modified ADSC-EVs through a circRNA/miRNA/NF-κB pathway

**DOI:** 10.1186/s12951-022-01696-z

**Published:** 2022-11-19

**Authors:** Yuhua Gao, Ningning Mi, Ying Zhang, Xiangchen Li, Weijun Guan, Chunyu Bai

**Affiliations:** 1 Institute of Precision Medicine, Jining Medical University, No.133 Hehua Road, Jining, Shandong 272067 People’s Republic of China; 2 Institute of Animal Sciences, Chinese Academy of Agricultural Sciences, No.2 Yuanmingyuan West Road, Haidian District, Beijing, 100193 People’s Republic of China; 3 College of Animal Science and Technology, College of Veterinary Medicine, Zhejiang A&F University, 666, Wusu Road, Li’an, Zhejiang, China

**Keywords:** PROM, MMPs, Macrophages, ADSCs, Extracellular vesicles

## Abstract

**Background:**

Circular RNA (circRNA) is a type of stable non-coding RNA that modifies macrophage inflammation by sponging micro RNAs (miRNAs), binding to RNA-binding proteins, and undergoing translation into peptides. Activated M1 phenotype macrophages secrete matrix metalloproteinases to participate in softening of the cervix uteri to promote vaginal delivery.

**Methods:**

In this study, the premature rupture of membranes (PROM) mouse model was used to analyze the role of macrophages in this process. Profiling of circRNAs was performed using a competing endogenous RNA microarray, and their functions were elucidated in vitro. Meanwhile, adipose tissue-derived stem cell-secreted extracellular vesicles (EVs) were applied as a vehicle to transport small interfering RNAs (siRNAs) targeting the circRNAs to demonstrate their biological function in vivo.

**Results:**

The miRNA *miR-1931* is dependent on the nuclear factor kappa-B (NF-κB) pathway but negatively regulates its activation by targeting the NF-κB signaling transducer TRAF6 to prevent polarization of M1 macrophages and inhibit matrix metalloproteinase (MMP) secretion. The host gene of circRNA *B4GALNT1*, also an NF-κB pathway-dependent gene, circularizes to form *circRNA_0002047*, which sponges *miR-1931* to maintain NF-κB pathway activation and MMP secretion in vitro. In the PROM model, EVs loaded with siRNAs targeting circRNAs demonstrated that the circRNAs reduced *miR-1931* expression to maintain NF-κB pathway activation and MMP secretion for accelerating PROM in vivo.

**Conclusions:**

Our data provide insights into understanding PROM pathogenesis and improving PROM treatment.

**Supplementary Information:**

The online version contains supplementary material available at 10.1186/s12951-022-01696-z.

## Introduction

Macrophages are phagocytic tissue leukocytes of the immune system that have differentiated from blood mononuclear cells and are located in almost all tissues in mammals. At the maternal–fetal interface, large amounts of leukocytes infiltrate into the pregnant endometrium. Rango et al. reported that, in the first trimester, almost 30% of all cells in the endometrium were leukocytes and that macrophages comprised 10% of all leukocytes [1]. Endometrial macrophages secrete many cytokines and chemokines at different stages of pregnancy. Interleukin (IL)-10, indoleamine 2,3-dioxygenase, and prostaglandin-E2 are secreted in early pregnancy to prevent maternal T-cell activation [2], and superoxide radicals and tumor necrosis factor (TNF)-α are secreted in late pregnancy to protect the fetus against infection and prevent preterm labor [3]. Meanwhile, matrix metalloproteinase (MMP) release is a secretory function of macrophages that accelerates collagen degradation to induce preterm or term delivery [4]. Thus, macrophages with different phenotypes have pivotal roles in the maintenance of pregnancy. Macrophages are polarized into M1 and M2 phenotypes according to their activation states, and the cytokines secreted by these macrophages have distinct pro- and anti-inflammatory roles. However, the molecular mechanisms involved in macrophage polarization and cytokine secretion during preterm birth remain unclear.

Circular RNAs (circRNAs) are a large class of non-coding RNAs that exhibit tissue- and stage-specific expression patterns in mammals [5–7]. They are more stable than linear RNA and are involved in different functions; hence, they are implicated in various diseases. Micro RNAs (miRNAs) are genome-encoded, small RNAs of 18–25 nucleotides in length that regulate gene expression through binding to the 3'-untranslated regions of target mRNAs to interfere with their expression. The circRNAs serve as a sponge to negatively regulate the expression of miRNAs through a widely reported competitive endogenous RNA mechanism. CDR1, a circRNA found in human brain tissue, has been demonstrated to function as a negative regulator that sponges the microRNA *miR-7*, further inhibiting its function [8]. Certain circRNAs have also been demonstrated to have a role in the polarization of macrophages. For example, *circRNA_09505* promoted polarization of M1 phenotype macrophages through sponging *miR-6089* to maintain activation of the AKT1/nuclear factor (NF)-κB signaling pathway in a gene-induced arthritis mouse model [9]. In atopic dermatitis, *circ_0004287* reduced the stability of metastasis-associated lung adenocarcinoma transcript 1 (MALAT1) by competitively binding to insulin-like growth factor 2 mRNA-binding protein 3, with MALAT1, in an N6-methyladenosine (m6A)-dependent manner to inhibit M1 phenotype macrophage activation [10]. These data prompted us to determine whether circRNA participates in the polarization of macrophages in preterm birth in vivo.

Extracellular vesicles (EVs) are endosome-derived vesicles of 50–200 nm in diameter that display “dish” shapes and are released by numerous cell types, including stem cells [11, 12] and immunocytes [13]. EVs play an important role in cell-to-cell communication. Maternal cells secrete and transport bioactivators, including mRNAs, non-coding RNAs, and proteins, which are transferred into recipient cells to influence cellular functions such as proliferation and apoptosis. Meanwhile, EVs with modified surfaces serve as drug-delivery vehicles to transport exogenous genes or chemical compounds for the treatment of various diseases [11]. In this study, we initiated lipopolysaccharide (LPS)-induced preterm birth in mice to investigate the role of macrophage polarization in premature rupture of membranes (PROM). A competing endogenous RNA (ceRNA) microarray was performed to analyze the interactions of ceRNAs in the endometrium to select potential target sites that influence macrophage polarization. EVs secreted by human adipose tissue-derived stem cells (ADSCs) were modified on their surface and then used as targeted delivery vehicles to treat premature fetal membrane rupture.

## Results

### Macrophage recruitment and polarization into the M1 phenotype in PROM mice

**Fig. 1:**
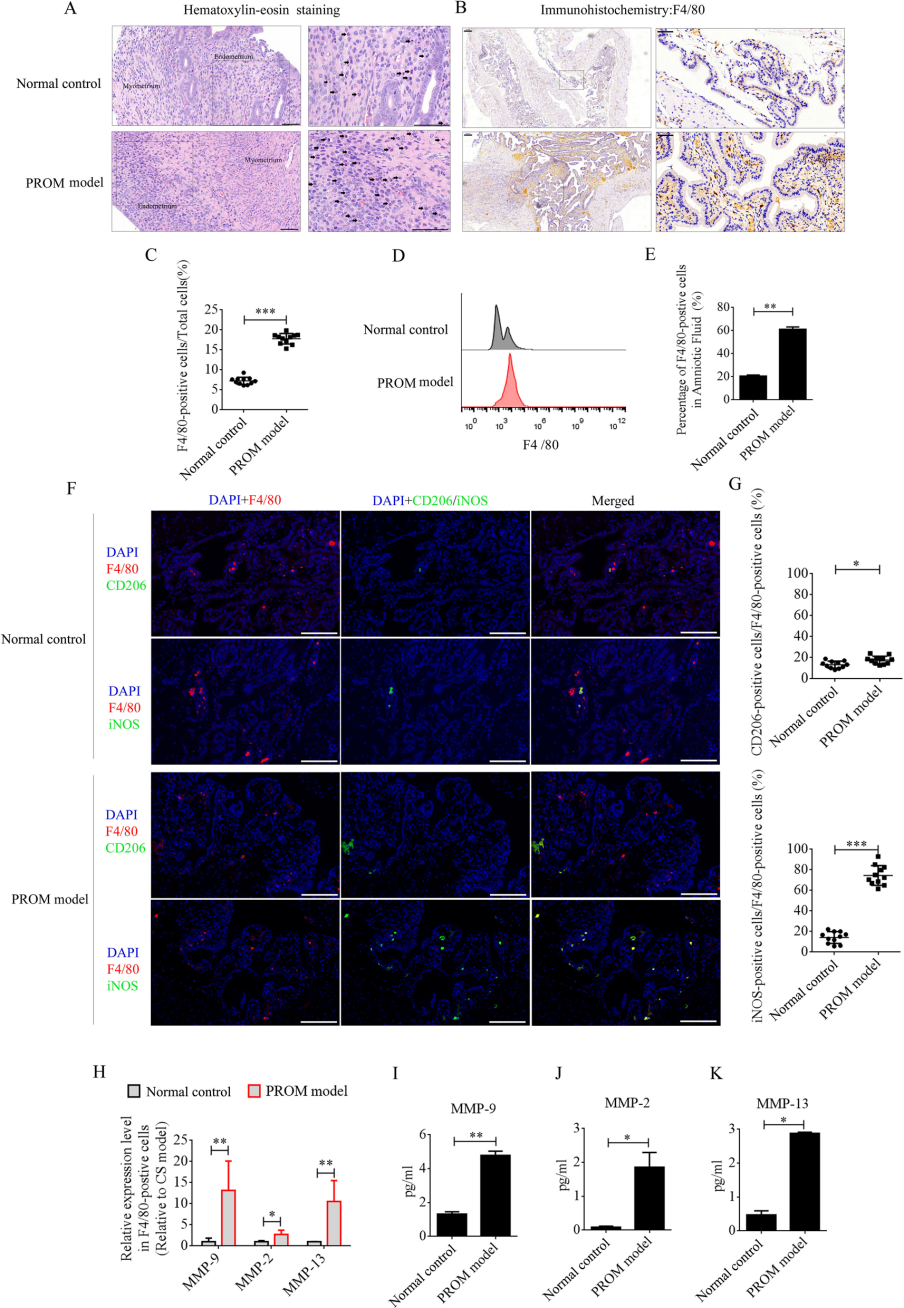
Recruitment and polarization of macrophages in the uterus of PROM model and normal control mice. **A** HE staining to analyze monocyte numbers in the same period in gestational uterus derived from normal control and PROM model mice. Representative monocytes are indicated with black arrows. **B** and **C** Anti-F4/80 antibody assessment and quantification of the number of macrophages in uteri. **D** and **E** Flow cytometry and counting of macrophages in amniotic fluid. **F** Representative immunofluorescence images of iNOS- or CD206-positive cells in the uteri. **G** Quantification of iNOS- and CD206-positive cells in the uteri. Scale bar = 100 μm. **H** mRNA levels of MMPs in F4/80-positive cells derived from normal control and PROM model mice. **I**, **J** and **K** ELISA detection of MMP-9, MMP-2, and MMP-13 expression in amniotic fluid; *p < 0.05; **p < 0.01; ***p < 0.001

LPS was intraperitoneally injected into mice at day 15 of pregnancy to establish the PROM model. PROM occurred at approximately 48 h. Maternal macrophages are gradually enriched in the decidua as pregnancy progresses [12]. LPS, as a classical inflammatory inducer of macrophages, promotes polarization to the M1 phenotype. A large number of inflammatory factors are then produced and secreted from the M1 macrophages, including MMPs, which accelerate collagen degradation and cervix softening to induce PROM. In this study, we first compared the recruitment and polarization of macrophages in uterine tissues and amniotic fluid between PROM model and pregnant normal control mice at day 17 of pregnancy. Under hematoxylin–eosin (HE) staining, monocytes were observed in the decidua of the uterus in both the PROM and normal control mice, and the numbers were found to be dramatically elevated compared with those in PROM model (Fig. 1A). F4/80 is the main marker of macrophages in different tissues, and immunohistochemistry (IHC) of uterine tissue revealed that F4/80-positive cells were expressed at significantly higher levels in the PROM model compared with those in the normal control (p < 0.001, Fig. 1B and 1C). Furthermore, the percentage of F4/80-positive cells in amniotic fluid was detected by flow cytometry. In agreement with the IHC data, the percentage of F4/80-positive cells in amniotic fluid from the PROM model was higher than in that from the normal control (p < 0.01, Fig. 1D and 1E). Next, F4/80-positive cells in uteri from PROM and normal control mice were analyzed by immunofluorescence for M1 or M2 phenotype polarization using inducible nitric oxide synthase (iNOS) or CD206 antibodies. The data revealed that almost 80% of F4/80-positive cells in the PROM model expressed iNOS, which was significantly higher than in the normal control (p < 0.05, Fig. 1F and G). These data suggested that M1 macrophages play a critical role in PROM. Next, F4/80-positive cells in amniotic fluid were selected by magnetic cell separation and used to detect mRNA expression of MMP genes by real-time PCR. Expression levels of *Mmp9*, *Mmp2*, and *Mmp13* mRNA were dramatically changed in F4/80-positive cells derived from the PROM model compared with those in cells from the normal control (Fig. 1H). Protein expression of MMP-9, MMP-2, and MMP-13 in amniotic fluid was analyzed by enzyme-linked immunosorbent assay (ELISA), which revealed that the levels were markedly elevated in the PROM model (p < 0.05, Fig. 1I–K).

### CircRNA acting as a miRNA sponge maintains polarization of macrophages and MMP secretion

**Fig. 2:**
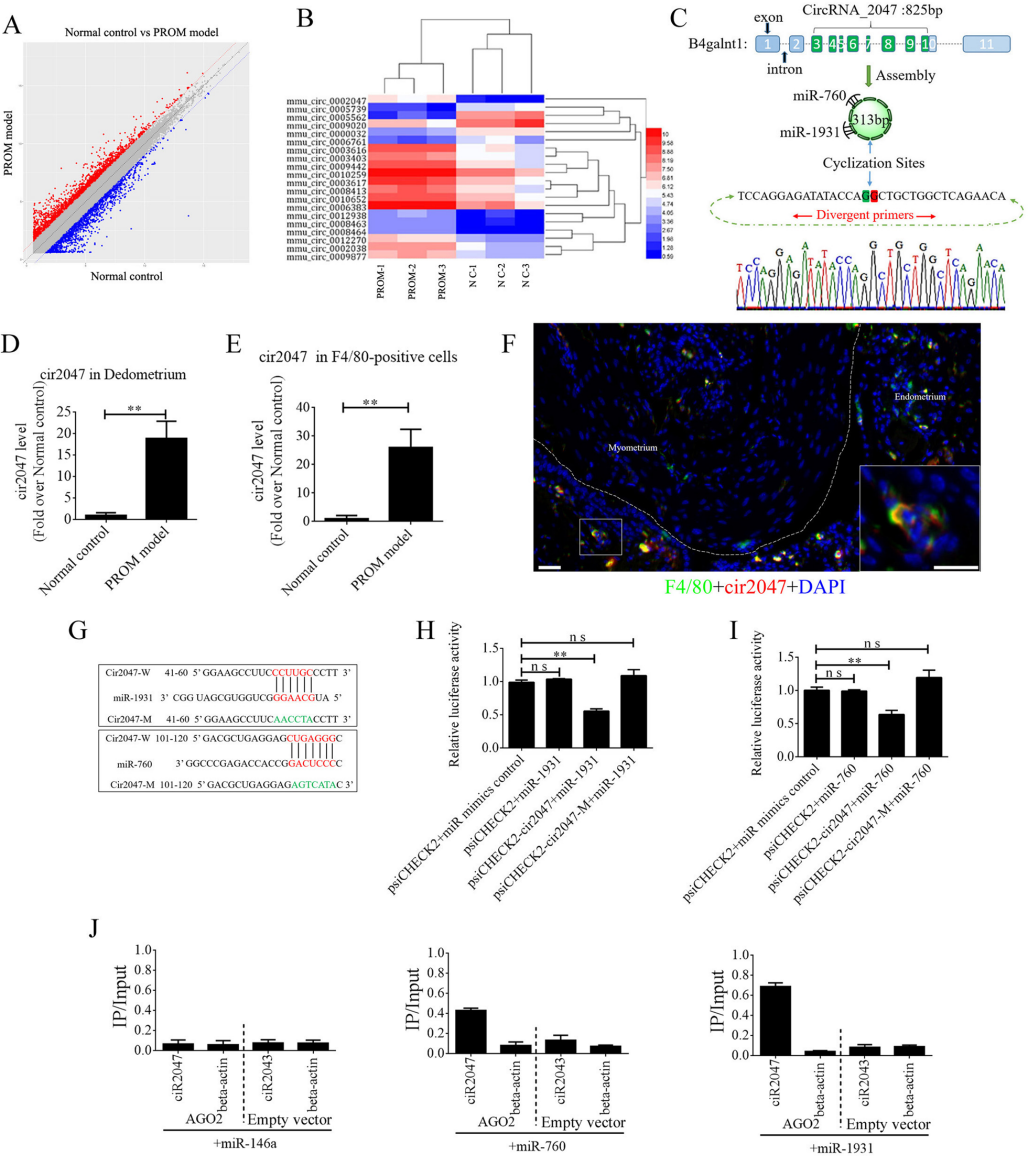
Changes in CircRNAs in the uteri of normal control (NC) and PROM model mice. **A** CeRNA profiling showing significant changes in circRNA expression in the uteri between NC and PROM model mice. **B** Heat map of the top 20 circRNAs showing significant changes following PROM occurrence. **C** Schematic representation of *cir2047* structure and sponged *miR-760-5p* and *miR-1931*. **D** and **E** Expression of cir2047 measured by qPCR in the decidua and F4/80-positive cells. **F** Co-expression of F4/80 and *cir2047* in uterine tissue detected by in situ hybridization and IF. Scale bar = 50 μm. **G** Binding sites of *cir2047* and miRNAs were predicted by bioinformatics algorithms and subsequently mutated to verify interactions. **H** and **I** Effects of *cir2047*-sponged *miR-1931* and *miR-760* were validated using luciferase reporter vector assays. Full-length *cir2047* and sequences containing different mutated variants of the miRNA-binding site (cir2047-M) were used to detect the suppressed effects of *miR-1931* and *miR-760*. **J** IP of AGO2 from F4/80-positive cells co-transfected with AGO2 and either *miR-1931*, *miR-760*, or *miR-146a* (negative control). Empty vector served as the AGO2-related negative control. *Cir2047* and *ACTB* mRNA levels were quantified by qPCR and relative IP/input (total cellular RNA) values were plotted; **p < 0.01; ns, not significant

The ceRNAs are widely reported to function in many biological processes and in disease occurrence. To exclude the underlying mechanism of ceRNA in maintaining polarization of macrophages, we peeled the decidua from the uterine lining and isolated total RNA to perform ceRNA profiling. Approximately 1000 circRNAs exhibited a significant change in their expression level between the PROM model and normal control (Fig. 2A). A heat map of the top 20 circRNAs is shown in Fig. 2B. Bioinformatics analysis revealed that mmu_circRNA_0002047 (*cir2047*, ENSMUST00000006914) has potential binding sites for *miR-1931* and *miR-760*, and was ranked among the most significantly changed genes in the analysis. *Cir2047* has eight exons covering 313 bp and is expressed by the host gene *B4galnt1*. To verify the ceRNA profiling data, we first confirmed a significant change in the level of *cir2047* in the decidua by examining F4/80-positive cells from amniotic fluid, before and after LPS treatment, using real-time PCR and product sequencing. The level of *cir2047* was elevated dramatically after LPS treatment (p < 0.01, Fig. 2C–E). To confirm *cir2047* expression in naïve macrophages in the uterus, in situ hybridization and IHC were performed following LPS treatment. The results demonstrated that *cir2047* and F4/80 were co-expressed in naïve macrophages (Fig. 2F). To investigate the interaction of *cir2047* and *miR-1931* or *miR-760*, the miRNA binding sites in *cir2047* were mutated by PCR (cir2047-mut) and then cloned into a luciferase reporter system, along with wild-type *cir2047* (cir2047-wt) as a positive control. These constructs were co-transfected into F4/80-positive cells with either a miRNA progenitor or a control. In F4/80-positive cells co-transfected with the miRNA progenitor and cir2047-wt, luciferase activity was dramatically decreased relative to cells co-transfected with the miRNA progenitor and cir2047-mut (p < 0.05, Fig. 2G–I). Argonaute 2 (AGO2) protein is an important component of the RNA-induced silencing complex that plays a central role in miRNA silencing. To confirm whether AGO2 serves as a binding platform for *miR-1931* and *cir2047*, we performed AGO2 immunoprecipitation (IP) in F4/80-positive cells carrying either an AGO2 expression vector or empty vector, and transiently co-expressed *miR-1931*, *miR-760*, or *miR-146a-5p* as a negative control (there is no *miR-146a-5p* binding site in the *cir2047* sequence). Real-time PCR was used to analyze the level of *cir2047* in IP products. *Cir2047* was specifically enriched by more than six- and four-fold in the presence of AGO2 in *miR-1931*- and *miR-760*-transfected cells, respectively, compared with that in the negative control (*miR-146a*-transfected cells; Fig. 2J).

**Fig. 3:**
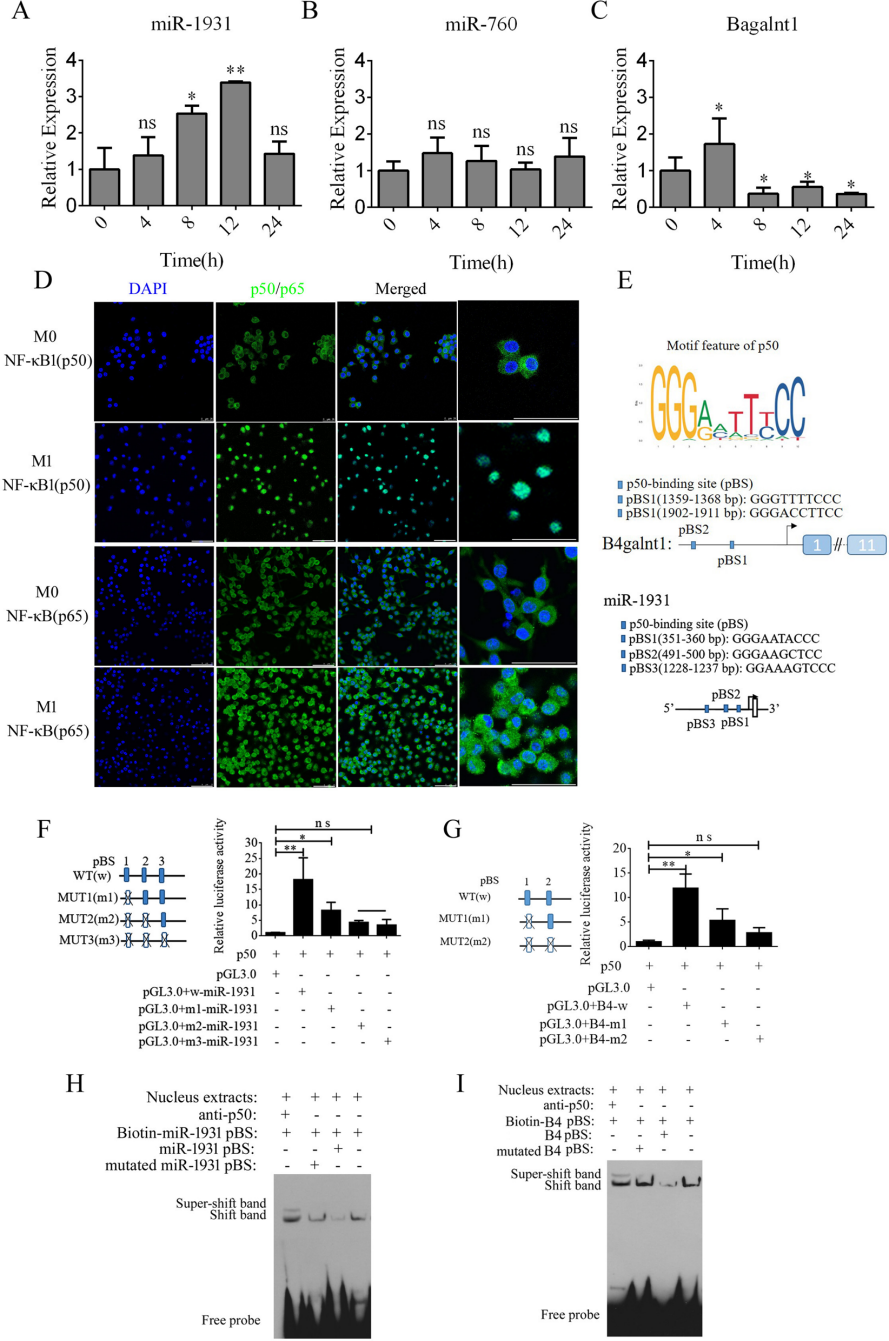
NF-κB pathway enhancement of transcription of *B4galnt1*, the host gene of *cir2047*, and *miR-1931* in macrophages. **A**–**C** Expression of *cir2047*, *miR-1931*, and *miR-760* measured by qPCR in F4/80-positive cells at different time points after polarization. **D** Immunofluorescence analysis of NF-κB (p65) and NF-κB1 (p50) in F4/80-positive cells after M1 polarization. **E** Schematic of each NF-κB1 (p50)-binding site (pBS) in the promoter regions of *B4galnt1* and *miR-1931*. **F** and **G** Effect of NF-κB1 (p50) on *B4galnt1* and *miR-1931* transcription through binding to their promotors was evaluated by luciferase reporter assays. **H** and **I** EMSA of physical binding of NF-κB1 (p50) to the *B4galnt1* and *miR-1931* promotor regions; *p < 0.05; **p < 0.01; ns, not significant

By analyzing the expression of *B4galnt1*, *miR-1931*, and *miR-760* in F4/80-positive cells using real-time PCR, we found that *B4galnt1* and *miR-1931* were transiently upregulated following LPS and interferon (IFN)-γ treatment in F4/80-positive cells, but *miR-760* did not undergo a significant change (Fig. 3A–C). These data implied that the linear mRNA of *B4galnt1* was circularized to form a circRNA that sponged *miR-1931* in F4/80-positive cells after LPS and IFN-γ treatment. The NF-κB pathway plays an important role in the polarization of M1 macrophages and in the production of pro-inflammatory factors. NF-κB is a dimer of the Rel family of five proteins, and a heterodimer of NF-κB (p65) and NF-κB1 (p50) subunits was the first described NF-κB molecule. We detected NF-κB (p65) and NF-κB1 (p50) by immunofluorescence in F4/80-positive cells following M1 polarization. In Fig. 3D, NF-κB1 (p50) had almost entered the nucleus, while some NF-κB (p65) remained in the cytoplasm. Bioinformatics analysis of the promoter regions of *B4galnt1* and *miR-1931* revealed that they are NF-κB pathway-dependent genes with promotor regions that bind NF-κB1 (p50) (Fig. 3E). To test the regulation of binding sites by NF-κB1 (p50), the binding sites for NF-κB1 (p50) were mutated individually or in combination through site-directed mutagenesis of the pGL3.0-B4galnt1 and pGL3.0-miR-1931 promotor vectors. We co-transfected the wild-type promotors of *B4galnt1* and *miR-1931* or their mutated (MUT) counterparts together with NF-κB1 (p50) overexpression vectors into HEK293T cells. The results revealed that NF-κB1 (p50) bound to sequences within the *B4galnt1* and *miR-1931* promoters to enhance their transcription (Fig. 3F and G). To further confirm whether NF-κB1 (p50) directly binds to the promotor regions of *B4galnt1* and *miR-1931* to influence their transcription, electrophoretic mobility shift assay (EMSA) was used to demonstrate the physical interactions between NF-κB1 (p50) and the promotor regions. The probes were designed for NF-κB1 (p50) according to its binding sites within the promoter sequence, and the results demonstrated that NF-κB1 (p50) did indeed bind to the respective binding sites. Super-shift bands were also clearly observed after incubation with antibodies against NF-κB1 (p50) (Fig. 3H and I).

**Fig. 4:**
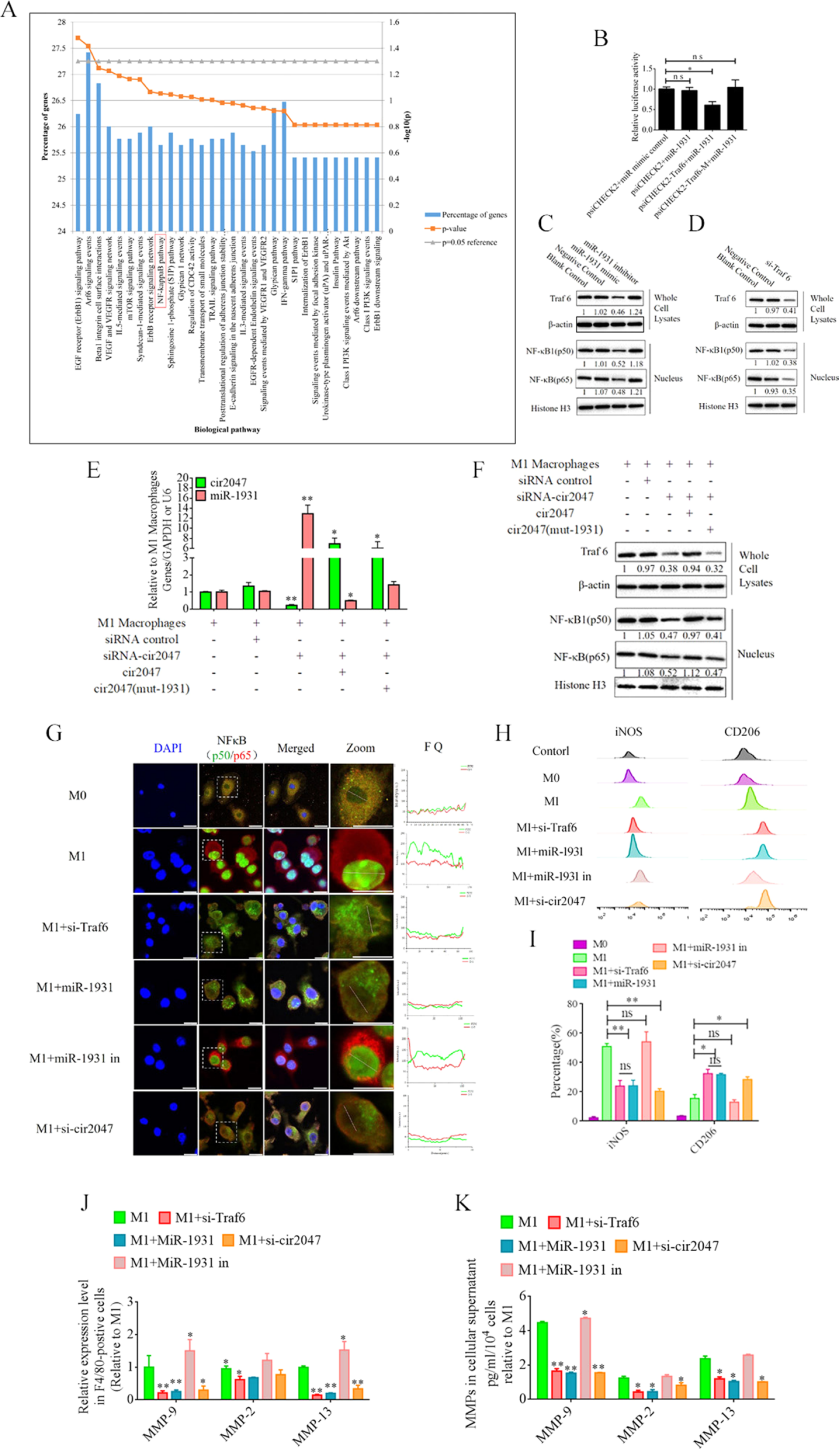
*Cir2047* maintains activation of the NF-κB pathway and the secretion of MMPs through sponging *miR-1931* in F4/80-positive cells. **A** The top 30 biological pathways, including the NF-κB pathway, generated by the predicted targets of *miR-1931*. **B** The effects of *miR-1931* on TRAF6 expression were validated using luciferase reporter assays. The 3'-UTR of *Traf6* and sequences containing mutated variants of miRNA-binding sites (M) were examined to determine their effect on miRNA expression. Results are expressed as relative luciferase activity and represent the mean ± standard deviation of at least three replicates. **C** Western blot showing TRAF6 expression levels in F4/80-positive cells at 48 h after transfection with specific siRNAs. The levels of NF-κB (p65) and NF-κB1 (p50) in nuclei were also decreased when *Traf6* was knocked down. **D** Western blot showing that *miR-1931* decreased the expression of NF-κB (p65) and NF-κB1 (p50) in the nucleus by targeting TRAF6 following treatment with an miRNA mimic. The relative density of bands is shown under each immunoblot after normalization to β-actin or histone levels. Representative blots from three independent experiments are shown. **E** qPCR showing *miR-1931* levels in F4/80-positive cells after transgenesis. **F** Western blotting revealed expression of TRAF6 in cells and NF-κB (p65) and NF-κB1 (p50) in nuclei after transgenesis. The relative band density is shown under each immunoblot after normalization to β-actin or histone levels. Representative blots from three independent experiments are shown. **G** Immunofluorescence detected the activation of the NF-κB pathway in F4/80-positive cells after transgenesis. Fluorescence images used to evaluate the localization of FITC-labeled NF-κB1 (p50) and Cy5-labeled NF-κB (p65); FQ, fluorescence quantitation; a.u., arbitrary unit. **H** and **I** Flow cytometry of M1 (iNOS) and M2 (CD206) macrophage markers in F4/80-positive cells after transgenesis and quantification of iNOS and CD206 expression levels in F4/80-positive cells (n = 3). **J** mRNA levels of *Mmp9*, *Mmp2*, and *Mmp13* in F4/80-positive M1 polarized cells after transgenesis. **K** ELISA of MMP-9, MMP-2, and MMP-13 expression in cellular supernatants derived from F4/80-positive M1 polarized cells after transgenesis; *p < 0.05; **p < 0.01; ns, not significant

To illustrate the role of *cir2047* in the polarization of M1 macrophages, we analyzed the potential function of *miR-1931* targets using the Functional Enrichment analysis tool (FunRich, http://www.funrich.org/). The top 30 biological pathways of *miR-1931* targets are shown in Fig. 4A. The predicted target of *miR-1931*, TRAF6, is a mediator of the NF-κB pathway and plays an important role in its activation. The small interfering RNA (siRNA) si-Traf6 was designed and applied to M1 macrophages to demonstrate that the levels of NF-κB1 (p50) and NF-κB (p65) were reduced dramatically following knockdown of *Traf6* by siRNA (p < 0.05, Fig. 4C). MiRNAs are important regulators of various biological processes that act through binding to the 3'-untranslated regions (UTRs) of targets to modulate their expression. Bioinformatics algorithm prediction, luciferase reporter assays, and western blotting revealed that *miR-1931* bound to the 3'-UTR of *Traf6* and regulated its expression (p < 0.05, Fig. 4B and D). *MiR-1931* repressed TRAF6 expression in the cytoplasm, downregulating the levels of p50 and p65 in the nucleus, while the opposite results were observed after the addition of *miR-1931* inhibitor (Fig. 4D). To further examine the role of *cir2047* in NF-κB pathway activation, specific siRNAs directed against cir2047-wt and cir2047-mut were transfected into M1 macrophages to analyze the RNA levels of *cir2047* and *miR-1931* and the protein levels of NF-κB1 (p50) and NF-κB (p65) in the nucleus. The data revealed that *cir2047* directly sponged *miR-1931* to maintain activation of the NF-κB pathway (Fig. 4E and F). Fluorescein isothiocyanate (FITC)-labeled NF-κB1 (p50) and Cy5-labeled NF-κB (p65) were used to evaluate localization. FITC and Cy5 fluorescence signals were initially observed in the nuclei of M1 macrophages, but after treatment with si-Traf6, *miR-1931*, or si-cir2047, the FITC signals were co-localized with Cy5 in the cytoplasm. Fluorescence quantitation of arbitrary single cells revealed lower FITC and Cy5 signals in the groups with si-Traf6, *miR-1931*, or si-cir2047 treatment compared with the signals in the group with M1 macrophages (Fig. 4G). Immunofluorescence showed that NF-κB1 (p50) and NF-κB (p65) were effectively restricted to the cytoplasm after knockdown of *Traf6* by siRNA, *miR-1931*, or si-cir2047. The expression rates of iNOS, a marker of M1 macrophages, and CD206, a marker of M2 phenotype macrophages, were further analyzed by flow cytometry. The results demonstrated that the percentage of CD206-positive cells in the groups treated with si-Traf6, *miR-1931*, or si-cir2047 were significantly higher than that in the non-treatment group (Fig. 4H and I). Next, the mRNA expression levels of *Mmp9*, *Mmp2*, and *Mmp13* were detected in cell supernatants of M1 macrophages. Treatment with si-Traf6, *miR-1931*, and si-cir2047 effectively decreased the expression levels of all three mRNAs (Fig. 4J and K). These data suggested that TRAF6, a target of *miR-1931*, had stabilized expression to maintain M1 polarization of macrophages through sponging by *cir2047* to secrete MMPs in inflammation reactions.

### EVs as a vehicle to transport si-Traf6, *miR-1931*, and si-cir2047 into macrophages

**Fig. 5:**
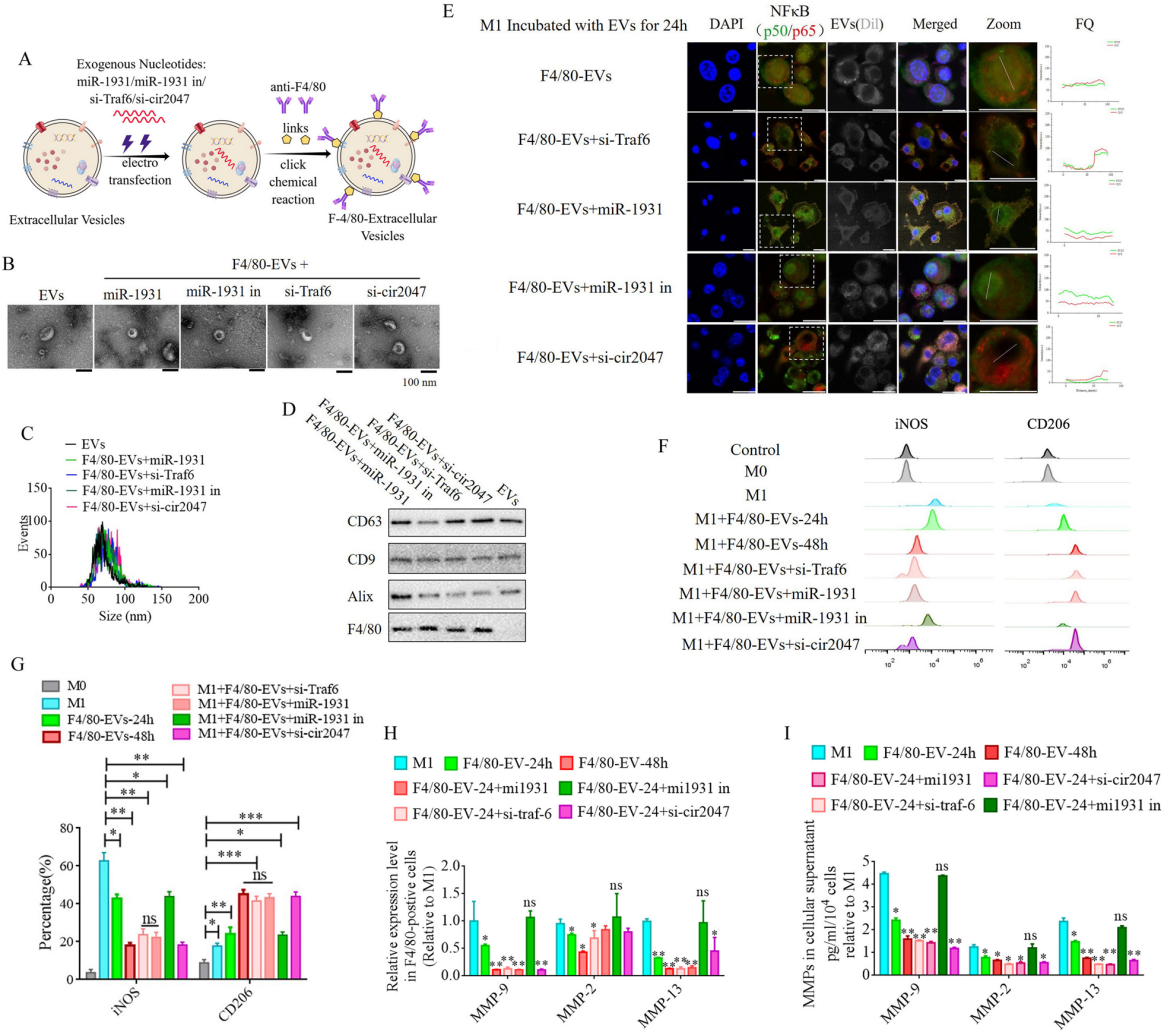
ADSC-secreted EVs as a vehicle to transport si-TRAF6, *miR-1931*, and si-cir2047 into macrophages in vitro. **A** Schematic illustration showing that siRNAs were loaded into ADSC-secreted EVs by electroporation. **B**–**D** Biological characteristics of these EVs, including morphology, particulate size distribution, and EV marker proteins. **E** Fluorescent images of unloaded F4/80-EVs and si-RNA-loaded F4/80-EVs incubated with M1 macrophages for 24 h were used to evaluate the localization of FITC-labeled NF-κB1 (p50) and Cy5-labeled NF-κB (p65). Fluorescence quantitation of arbitrary single cells revealed lower FITC and Cy5 signals in the group that received siRNA-loaded F4/80-EVs compared with the signals in the group with unloaded F4/80-EVs; FQ, fluorescence quantitation; a.u., arbitrary unit. **F** and **G** Flow cytometry of M1 (iNOS) and M2 (CD206) macrophage markers after treatment with various F4/80-EVs and quantification of iNOS and CD206 expression levels in various F4/80-EV-treated macrophages (n = 3). **H** mRNA levels of *Mmp9*, *Mmp2*, and *Mmp13* in F4/80-positive M1 polarized cells after treatment with various F4/80-EVs. **I** ELISA of MMP-9, MMP-2, and MMP-13 expression in cellular supernatant derived from F4/80-postive M1 polarized cells after treatment with various F4/80-EVs; *p < 0.05; **p < 0.01; ***p < 0.001; ns, not significant

To further analyze the roles of *cir2047*, TRAF6, and *miR-1931* in the polarization of macrophages in vitro and in vivo, EVs from human ADSCs were applied as a vehicle to transport siRNAs against *cir2047*, *TRAF6* and *miR-1931,* or *miRNA-1931* mimic, to prevent M1 polarization in macrophages. First, the biological characteristics of human ADSCs were identified, including specific markers and multi-differentiation potential. These results demonstrated that CD29, CD90, and CD105 were positive in human ADSCs, and that the ADSCs had successfully differentiated into chondrocytes, adipocytes and osteoblasts (Additional file 1: Figure S1). Previous reports [13, 14] demonstrated that EVs derived from mesenchymal stem cells carry certain miRNAs and proteases to reverse the dominant phenotype from M1 to M2 in macrophages. In this study, 1 × 10^8^ particles/mL ADSC-EVs were labeled with Dil and then incubated with M1 macrophages. The signal intensities of FITC-labeled NF-κB1 (p50) and Cy5-labeled NF-κB (p65) in the nucleus were used to assess the activation of the NF-κB pathway after 24-h and 48-h treatment. However, despite the signal being slightly reduced at 24 h, the signal level had completely diminished to the same level observed in M0 macrophages after the 48-h treatment (p < 0.05, Additional file 1: Figure S2). Next, EVs were loaded with siRNAs against *cir2047*, *Traf6*, and *miR-1931* using electroporation. To improve the delivery efficiency of these EVs in vivo, a rat monoclonal antibody against F4/80 was bound to the surface of EVs using click chemistry (F4/80-EVs; Fig. 5A). First we compared the internalization efficiencies of F4/80-EVs and normal ADSC-EVs into recipient M1 macrophages. Dil-labeled EVs (1 × 10^8^ particles/mL) incubated with M1 macrophages for 24 h revealed that the fluorescence level was significantly higher in F4/80-EV-treated than in normal EV-treated M1 macrophages, implying that F4/80-EVs were internalized more efficiently than normal EVs (p < 0.05, Additional file 1: Figure S3).

Normal EVs and F4/80-EVs loaded with *miR-1931* (F4/80-EVs + miR-1931), si-Traf6 (F4/80-EVs + si-Traf6), miR-1931 inhibitor (F4/80-EVs + miR-1931 in) or si-cir2047 (F4/80-EVs + si-cir2047) were then analyzed for morphology, size, and characteristic EV marker proteins. Transmission electron microscopy (TEM) showed that normal EVs and F4/80-EVs loaded with different siRNAs displayed a round shape and were intact, with no membrane damage (Fig. 5B). Nanoparticle tracking analysis revealed that the mean diameter sizes of normal EVs, F4/80-EVs + miR-1931, F4/80-EVs + si-Traf6, F4/80-EVs + miR-1931 in, and F4/80-EVs + si-cir2047 were similar (88.32 ± 9.47, 86.44 ± 10.54, 91.35 ± 7.61, 87.59 ± 8.74, and 90.18 ± 9.47 nm, respectively, Fig. 5C), and were within the size range of known exosomes (50–200 nm). Western blotting indicated that characteristic EV marker proteins CD63, CD9, and Alix were positive in all EVs, but F4/80 was not expressed in normal EVs (Fig. 5D).

Next, M1 macrophages were incubated with these EVs for 24 h, and immunofluorescence and flow cytometry were used to analyze NF-κB pathway activation and reversed macrophage polarization. The results indicated that EVs loaded with these siRNAs could effectively reduce the signal intensity of FITC-labeled NF-κB1 (p50) and Cy5-labeled NF-κB (p65) in the nucleus after 24 h, but excluded loaded *miR-1931* mimic (Fig. 5E). Flow cytometry data demonstrated that the percentage of CD206-positive cells in the group receiving F4/80-EVs loaded with siRNAs was significantly higher than that in the F4/80-EV group after 24 h, which was also observed in the F4/80-EV group after 48 h (p < 0.05, Fig. 5F and G). Furthermore, EVs loaded with si-Traf6, *miR-1931*, or si-cir2047 effectively decreased the mRNA expression of *Mmp9*, *Mmp2*, and *Mmp13* in M1 macrophages after 24 h (p < 0.05, Fig. 5H), and protein expression of MMP-9, MMP-2, and MMP-13 was also declined in cellular supernatants (p < 0.05, Fig. 5I). The downtrend of MMP-9, MMP-2, and MMP-13 after 24 h in the groups treated with siRNA-loaded F4/80-EVs remained in step with those treated with F4/80-EVs at 48 h. In previously study reported that the exosomes derived from M2 macrophages inhibited tumor growth by reprogramming tumor-associated macrophages into M1-like macrophages [15], thus, the possibilities of the repolarization of the M2 to M1 phenotype was detected after treatment with these EVs in this research. To examine the effects of these EVs in M2 macrophages following a 48-h incubation, flow cytometry was used to detect potential reversed macrophage polarization. The results indicated that the percentages of iNOS-positive cells in the groups receiving siRNA-loaded F4/80-EVs were not significantly different from that in untreated M2 macrophages after 48 h (Additional file 1: Figure S4). These data implied that the F4/80-EV groups treated with siRNAs conferred the EVs with improved capacity to convert the macrophage phenotype from M1 to M2 in vitro.

### Targeted delivery of F4/80-EVs to macrophages in vivo and its therapeutic efficacy in PROM

**Fig. 6:**
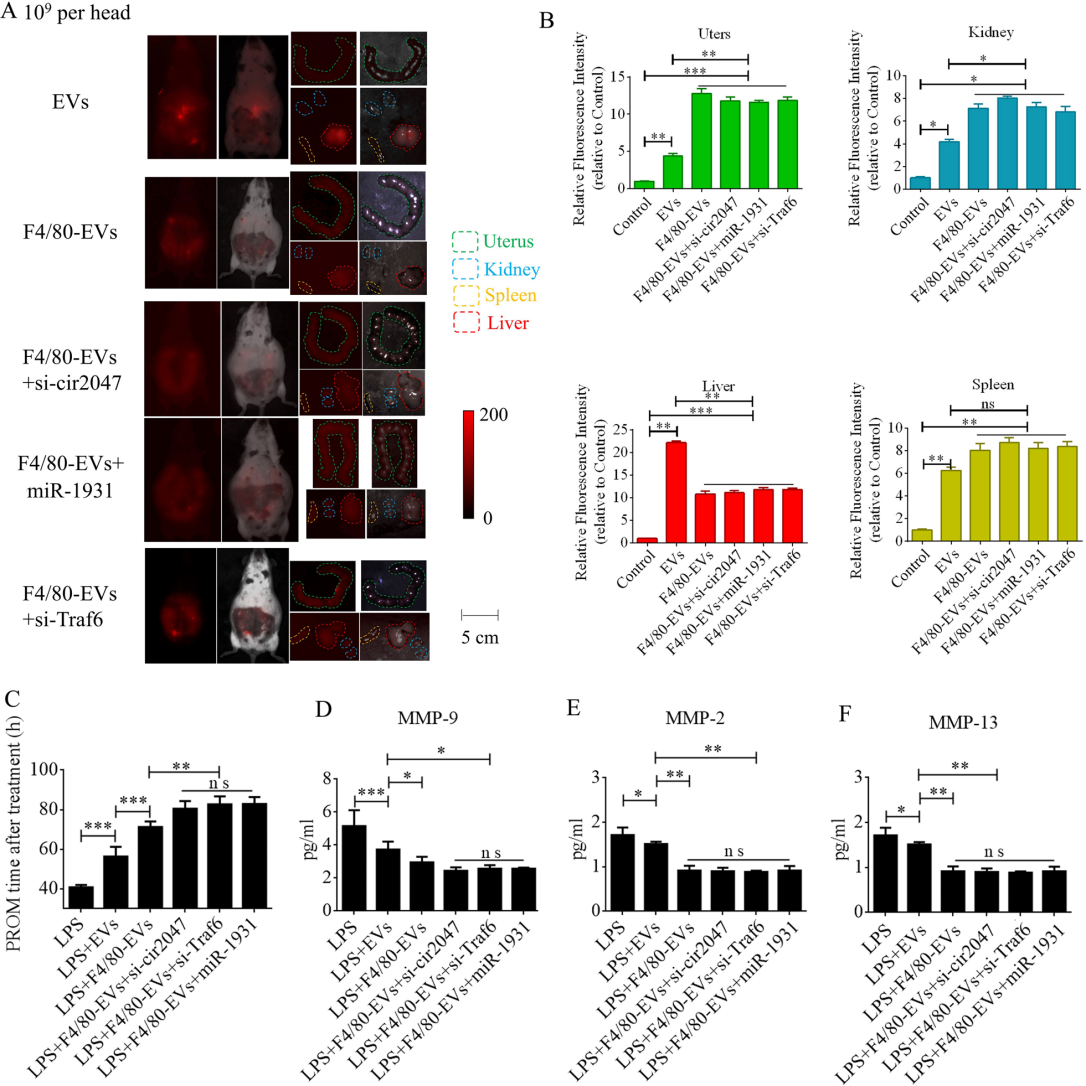
Therapeutic efficacies of different F4/80-EVs in PROM mice. **A** Representative fluorescence imaging of various F4/80-EVs (1 × 10^9^ particles/mouse) in whole-body and organ tissues from PROM mice. **B** Relative fluorescence intensity in the uterus, kidneys, liver, and spleen of PROM mice (n = 3). **C** Analysis of PROM timing after various F4/80-EV treatments in the PROM mouse model (n = 5). **D**–**F** ELISA of MMP-9, MMP-2, and MMP-13 expression in amniotic fluid derived from normal control and PROM model mice (n = 3); ns, not significant; *p < 0.05; **p < 0.01; ***p < 0.001

**Fig. 7:**
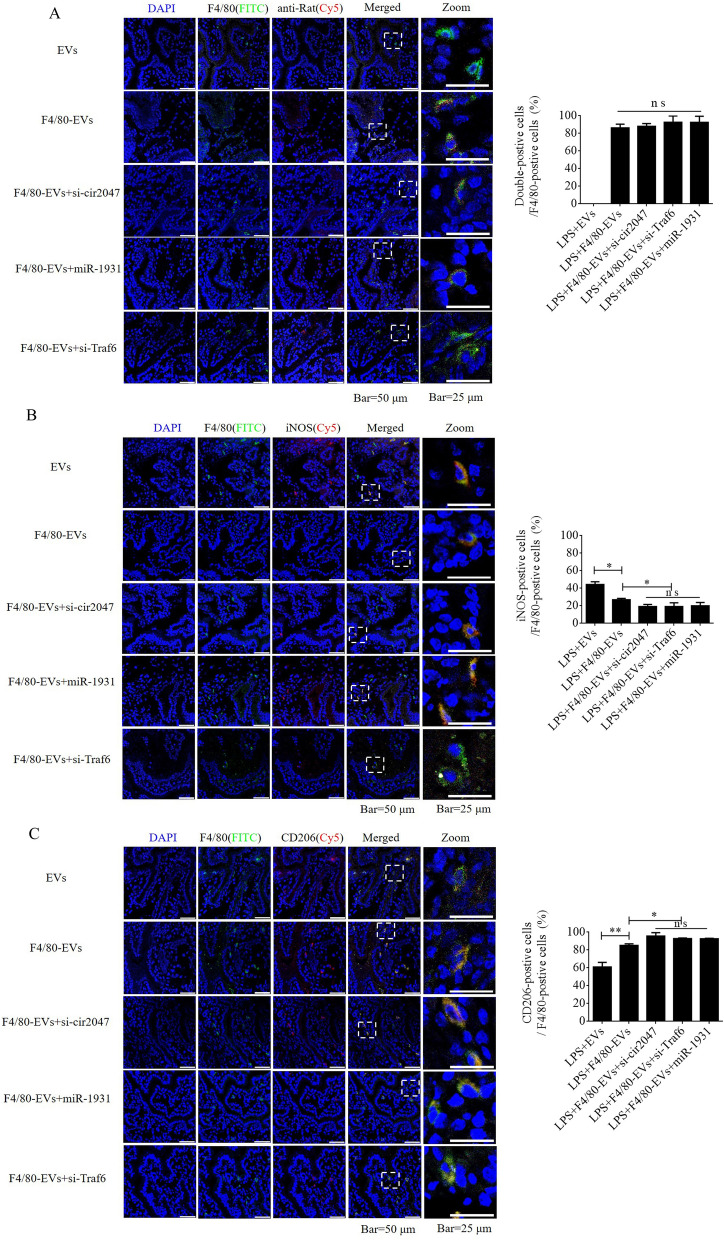
Targeted delivery of F4/80-EVs into macrophages in vivo. **A** Location of F4/80-EVs in uterine sections from F4/80-EV-treated PROM mice using the corresponding Cy5-labeled secondary antibody of F4/80. A rabbit polyclonal antibody against F4/80 labeled with FITC was used to verify macrophages. Double-positive cells indicated macrophages that carried F4/80-EVs (n = 3). **B** and **C** Representative immunofluorescence images of iNOS- and CD206-positive cells in the uteri of PROM mice treated with various F4/80-EVs and quantification of iNOS- and CD206-positive cells in uterine tissues following these treatments (n = 3); *p < 0.05; **p < 0.01; ***p < 0.001; ns, not significant

To further verify F4/80-mediated endocytosis of F4/80-EVs by macrophages, pregnant mice were used to assess targeted delivery of EVs, F4/80-EVs, or siRNA-loaded F4/80-EVs (1 × 10^9^ particles/mouse delivered by intrauterine injection) for the treatment of PROM. These EVs were labeled with DiR, and their location was analyzed by whole body fluorescence and organ fluorescence. Fluorescence signals were observed with EV treatment, but not with the blank control. Most fluorescence signals remained in the liver at 24 h after normal EV treatment, whereas the fluorescence signals had spread to the abdomen of mice treated with F4/80-EVs and siRNA-loaded F4/80-EVs, and the uteri showed stronger fluorescence signals than those in mice treated with EVs. Compared with EVs, F4/80-EVs and siRNA-loaded F4/80-EVs mostly accumulated in the uterus, with some distribution in the kidneys, spleen, and liver of pregnant mice (Fig. 6A). The fluorescence intensity of F4/80-EVs and siRNA-loaded F4/80-EVs in the uterus was significantly higher than that of EVs (Fig. 6B). Next, we analyzed the timing of PROM occurrence and found that it was extended after treatment with all EVs, but significant changes were observed in the groups receiving siRNA-loaded F4/80-EVs, including si-TRAF6, si-*cir2047*, and *miR-1931* mimic (Fig. 6C). Furthermore, the expression levels of MMP-9, MMP-2, and MMP-13 were markedly reduced in the amniotic fluid of pregnant mice after all EV treatments, with the most obvious effects observed in mice treated with siRNA-loaded F4/80-EVs (Fig. 6D–F). To analyze the potential toxicity of these EVs in mice, ELISA was used to detect the inflammatory factors TNF-α and IL-1β, the biochemical indicators of hepatotoxicity and nephrotoxicity, alanine transaminase (ALT), aspartate aminotransferase (AST), serum creatinine, and blood urea nitrogen. The data revealed that TNF-α, IL-1β, ALT, AST, serum creatinine, and blood urea nitrogen levels were significantly decreased in serum following treatment with these EVs in LPS-treated mice, which implied that there was no toxicity in vivo (Additional file 1: Figure S5). To verify the targeted delivery of F4/80-EVs to macrophages in vivo, FITC-labeled rabbit polyclonal antibody against F4/80 was used to locate macrophages in uterine sections, and Cy5-labeled anti-rat antibody was used to locate F4/80-targeted rat polyclonal antibody that was bound to the surface of EVs. Double-positive cells indicated that the macrophages contained these F4/80-EVs and confirmed that there were no significant changes in the rate of double-positive cells in the groups treated with F4/80-EVs and F4/80-EVs loaded with si-Traf6, si-cir2047, or *miR-1931* mimic (Fig. 7A). To investigate the effects of EVs and these F4/80-EVs on macrophage polarization and their therapeutic efficacy in vivo, iNOS-positivity and CD206-positivity were analyzed in F4/80-positive cells of uterine sections. The data revealed that the number of iNOS-positive cells was markedly declined, and almost 80% of F4/80-positive cells expressed CD206 after treatment with these F4/80-EVs. Furthermore, the effects were stranger after treatment with F4/80-EVs loaded with si-Traf6, si-cir2047, or *miR-1931* mimic (Fig. 7B and 7C). Taken together, our data indicated that *cir2047* maintained the polarization of M1 macrophages and promoted MMP secretion through the *miR-1931*/TRAF6/NF-κB pathway to accelerate PROM.

## Discussion

Macrophages play an important role in natural labor, secreting numerous MMPs and inflammatory cytokines, including IL-1β and TNF, which soften the cervix uteri and degrade the fetal membrane to promote vaginal delivery [16]. During degradation of the fetal membrane, small sterile ruptures (outer diameter < 0.47 mm) of the amniotic membrane promote epithelial–mesenchymal transition of alveolar epithelial cells, the healing of which is facilitated by IL-1β and TNF from recruited macrophages, but bigger ruptures (outer diameter > 0.91 mm) promote degradation of the fetal membrane through macrophage participation [17]. MMPs are a large family of zinc-dependent endopeptidases that can degrade all extracellular matrix components in physiological and pathological processes [18, 19]. The main mechanical properties of the cervix are derived from organization of the collagen network and extracellular matrix, which become disorganized after degradation by macrophage-secreted MMPs during softening of the cervix [20]. Macrophages have plastic characteristics that facilitate polarization into the M1 and M2 phenotypes. M1 macrophages are functionally pro-inflammatory and antimicrobial, while M2 macrophages are anti-inflammatory and are involved in tissue repair [21]. Inflammatory factors and MMPs are secreted by M1 macrophages when they are activated by pathological factors. Therefore, abnormally activated M1 macrophages destroy the structure of the cervix uteri and fetal membrane to initiate abortion during pregnancy. The NF-κB pathway is a canonical signaling pathway associated with inflammation, and almost all inflammatory factors are encoded by downstream genes. The core transcription factor of the NF-κB pathway is a dimer comprising members of the Rel family. A heterodimer of p65 and p50 subunits is inhibited by IκB protein in unstimulated cells. IκB is a Rel family member that binds to NF-κB, restricting it to the cytoplasm and inhibiting its activity. Phosphorylation of IκB by the IKK complex marks IκB for degradation through a ubiquitin-dependent pathway in the proteasome, which liberates NF-κB to enter the nucleus, bind to DNA, and activate transcription. TRAF6, a member of the TRAF family and an E3 ubiquitin ligase, is a signal transduction molecule shared by the IL-1 receptor/TLR family and the TNF receptor superfamily. Numerous reports demonstrated that TRAF6 plays an irreplaceable role in many pathophysiological processes by activating the NF-κB pathway [22, 23]. Other reports demonstrated that suppressed expression of TRAF6 resulted in lowering the phosphorylation levels of IκB to inhibit activation of the NF-κB pathway [24]. Several studies also demonstrated that miRNAs directly suppress TRAF6 at the post-transcriptional level to inhibit the activation of the NF-κB pathway [25, 26]. Taken together, TRAF6 is an important target for preventing the activation of the NF-κB pathway in macrophages, and its transcription can be regulated by miRNAs.

Recent reports indicated that non-coding RNAs (ncRNAs), including miRNA, circRNA, and long non-coding RNA, play important roles in the progression of development and occurrence of disease. CircRNAs are stable ncRNAs and have binding sites that sponge miRNAs to inhibit their biological function through the mechanism of ceRNA in autoimmune imbalance and the inflammatory response [27, 28]. Yang et al. demonstrated that *miR-6089* negatively regulated the NF-κB pathway through directly suppressing the expression levels of AKT to inhibit inflammation processes in macrophages, while circRNA_09505 positively regulated macrophage inflammation by functioning as a sponge to bind *miR-6089* in an arthritis model [9]. These reports implied that circRNAs influence inflammation reactions by regulating activation of the NF-κB pathway. Acting as a specific miRNA of macrophages following M1 phenotype polarization, *miR-1931* maintains a higher abundance of early-response M1 polarization, and shows decreased expression in M1 macrophages after 4 h of polarization, confirming its role as an M1 early-response miRNA [29]. Thus, the results of ectopic expression of *miR-1931* in M1 macrophages were unpredictable. Our study found that *miR-1931* directly bound the 3'-UTR of *Traf6* to inhibit its expression, thereby preventing NF-κB pathway activation. Interestingly, bioinformatics prediction and experimental verification identified *miR-1931* as an NF-κB pathway-dependent gene. To maintain NF-κB pathway activation and accelerate the polarization of M1 macrophages, *miR-1931* levels should be reduced, but this molecular mechanism remains unclear. B4GALNT1 is an essential glycosyltransferase for the synthesis of complex gangliosides, which play crucial roles in the maintenance of the integrity of the nervous system and in neurodevelopment [30–32]. CircRNAs modulate pathological processes by sponging miRNAs, binding to RNA-binding proteins, regulating splicing and transcription, modifying parental gene expression, regulating protein translation, or being translated into peptides. The main biological characteristic of a circRNA is the formation of a covalently closed-loop structure that is generated through a special type of alternative splicing termed “back-splicing” [33]. In our study, *B4galnt1* was also an NF-κB pathway-dependent gene with a promotor region that bound NF-κB 1(p50) and served as a host gene for mmu_circRNA_0002047, which had a binding site for sponging *miR-1931* and maintaining activation of the NF-κB pathway. However, the mechanism of the closed-loop structure formation and the enzymes involved in alternative splicing of the cyclic form of *cir2047* when polarized to M1 macrophages remains unknown.

In conclusion, we found that circRNA formation in uterine macrophages maintains NF-κB pathway activity and accelerates M1 phenotype polarization to upregulate MMP secretion for softening of the cervix uteri and degradation of the fetal membrane to further promote PROM. This mechanism serves as a sponge to reduce *miR-1931* expression, and *miR-1931* negatively regulated the NF-κB pathway through inhibiting the expression of NF-κB signaling transducer TRAF6, thereby forming a regulatory circuit.

## Materials and methods

### Ethics statement

This study was performed in strict accordance with recommendations from the Institutional Animal Care and Use Committee of Jining Medical University. All protocols were approved by the Committee on the Ethics of Animal Experiments of Jining Medical University (License ID: 2018-jc-019). The schematic illustration was drawn using Figdraw tools (www.figdraw.com).

### PROM model

Pregnant Kunming mice weighing 17–23 g were used in this study. The uterus and amniotic fluid were obtained from normal pregnant mice (approximately embryonic day 17) through caesarean section and used as normal controls. LPS was used to induce PROM as described previously [34]. LPS (100 μg/kg body weight) was injected intraperitoneally into mice at 15 days of pregnancy. PROM was detected after approximately 48 h of treatment. When bleeding was found in the vagina, the uterus and embryos were isolated under aseptic conditions, without releasing the amniotic fluid. The uterine tissues and amniotic fluid were analyzed for expression of specific genes and compared with those from caesarean sections. Hematoxylin–eosin (HE) staining was used to analyze pathological changes in uterine tissues derived from PROM model or normal control mice at day 17 of pregnancy.

### Isolation and culture of F4/80-positive cells

Amniotic fluid was centrifuged at 5000 rpm for 30 min to collect macrophages, amniotic fluid stem cells, fibroblasts, and other cells. The collected cells were cultured in DMEM (Gibco, USA) supplemented with 10% fetal bovine serum (FBS). The macrophages, F4/80-positive cells in amniotic fluid, were selected by magnetic cell separation and cultured in complete medium consisting of DMEM (Gibco, USA) supplemented with 10% FBS.

### Analysis of recruited macrophages in the uterus

Uteri were obtained from LPS-treated aborting mice and normal pregnancy mice, sectioned and stained with HE for histological analysis. F4/80, a macrophage marker, was used to mark and count macrophages in the uterus by immunofluorescence. Positive cells were counted and analyzed using ImageJ.

### Microarray analysis

Analyses of the Agilent Mouse miRNA (8 × 60 K) array and SBC Mouse (4 × 180 K) ceRNA array results were performed by the National Engineering Center for Biochip (Shanghai, China). All data were analyzed using the SBC Analysis System (online tools for analysis of microarrays, http://sas.ebioservice.com/portal/root/molnet_shbh/index.jsp) to identify differentially expressed genes [35]. The significance of differential expression was assessed by the Student’s t-test. Fold changes of ≥ 2 and p values of < 0.05 were used as the thresholds. Heat maps were generated using Java TreeView software.

### Luciferase reporter assay

Firefly luciferase reporter genes were constructed using the pCL3.0-Luc vector (Promega, Madison, WI, USA) and target sequences. The sequence was mutated at positions 3–5 of the seed sequence using a QuikChange mutagenesis kit (Stratagene, USA). The HEK293T cells were transfected using Lipofectamine 3000 (Invitrogen, USA) with a mixture of 1 mg/mL firefly luciferase reporter plasmid, 20 nM miRNA precursor or control, and 20 ng/mL *Renilla reniformis* luciferase-encoding plasmid (pRL-TK; Promega). Cells without the transfected precursor served as the control for normalization, and luciferase activity was measured at 48 h post-transfection using a dual luciferase assay system. All assays were repeated independently at least three times.

### Real-time PCR

MiRNAs were isolated using a miRcute miRNA isolation kit (Tiangen, Beijing, China). The cDNA was synthesized with a High Capacity RNA-to-cDNA Kit (Tiangen, Beijing, China). Real-time PCR was performed with SYBR PCR Mix (SYBR Green; Tiangen, Beijing, China) and primers for amplification of *miR-1931* and *miR-760-5p* (Tiangen Biotech CO., LTD.) in a Light Cycler 480 PCR system (Roche, USA). U6 small nuclear RNA was used for normalization. For mRNA, total RNA was extracted using Trizol reagent and reverse transcribed with sequence-specific reverse transcription-PCR primers (HiScript III RT SuperMix, Vazyme, China). PCR was performed using a ChamQ Universal SYBR quantitative PCR (qPCR) Master Mix (Vazyme, China). Specific primers for mRNAs and the endogenous control (β-actin) were synthesized by Sangon Biotech (Shanghai, China). Gene expression was detected on the Light Cycler 480 PCR system. Each experiment was performed in duplicate in 96-well plates and repeated three times. Primer sequences were as follows: B4galnt 1, F: 5'-CTTCACCATCCTCGTAAGACAC-3', R: 5' -AGTGTCCGTAGTCGATCATAAC-3'; MMP-2, F: 5'—TTCCCCAGAGTCTACGTGCT-3', R: 5'-CGGCACTAAGACCTAACTGGA-3'; MMP-9, F: 5'-CATCCTTAACGCAGTTGCT-3', R: 5'-ATCCTGATCTTCATCGAGGTGA-3'; MMP-13, F: 5'-TTCAGCTCCAATAAATTGACA-3', R: 5'-GTAATATACTCAACGCTGGT-3'.

### Validation of circRNAs

Total RNA from the decidua and F4/80-positive cells was reverse transcribed by random primers for real-time PCR assays performed using the Roche 480 real-time PCR system and specific primers to evaluate the abundance of circRNAs. Product sequencing was used to identify the dependability of PCR. CircRNAs were normalized to β-actin (Sangon Biotech, China). CircRNAs, F: 5'- CCAGGAGATATACCAGGCTGC-3', R: 5'-ATTCCTGCTCCCTGGCGACA-3'. For validation of circRNA expression in F4/80-positive cells, frozen sections of uterine tissue were subjected to in situ hybridization with a nucleic acid probe (5'Cy5-AGCAGCCTGGTATATCTCCT-3'Cy5). After the hybridization, FITC-labeled F4/80 antibody was added to highlight macrophages. After counterstaining with DAPI, images were obtained by confocal microscopy.

### CircRNA overexpression and silencing

Cir2047 (> mmu_circ_0002047|ENSMUST00000006914|B4galnt1) was as follows: GCTGCTGGCTCAGAACAACTGCAGTTGTGAATCCAAGGGAGGAAGCCTTCCCTTGCCCTTTCTGAGACAGGTTCGGGCGGTTGACCTCACTAAAGCCTTTGACGCTGAGGAGCTGAGGGCTGTTTCTGTCGCCAGGGAGCAGGAATACCAGGCCTTCCTTGCAAGGAGCCGGTCCCTGGCTGACCAGCTGCTGATAGCTCCCGCCAACTCCCCCTTACAGTACCCCCTGCAGGGTGTGGAGGTTCAGCCCCTCAGGAGCATCCTGGTGCCAGGGCTAAGTCTGCAGGAAGCTTCTGTCCAGGAGATATACCAG). The cir2047 overexpression vector was constructed with front and back circular frames containing the endogenous flanking genomic sequence with an *Eco*RI restriction enzyme site in the front circular frame, and a part of the inverted upstream sequence with a *Bam*HI site in the back circular frame. It was co-cloned with the cir2047 transcript into the pLCDH-ciR vector. Vector construction was verified by sequencing. This vector was constructed with support from Guangzhou Geneseed Biotech Co., Ltd (Guangzhou, China). A siRNA of cir2047 (si-cir2047, 5'- GGAGATATACCAGGCTGCT-3') was designed, synthesized, and used to assay the function of cir2047 after selection.

### Human ADSC culture and isolation of ADSC-derived EVs

Human ADSCs were purchased from Cyagen (Guangzhou, China) and cultured in medium designed for the growth of human adipose-derived mesenchymal stem cells (OriCell®, Cyagen). To analyze the biological characteristics of human ADSCs, their main markers, CD29, CD105 and CD90, were detected by flow cytometry, and chondrogenic, osteogenic, and adipocyte differentiation of human ADSCs was performed with specific medium (OriCell®, Cyagen). To obtain ADSC-derived EVs, OriCell® medium containing FBS without EVs was used to culture ADSCs. Freshly prepared culture supernatant was centrifuged at 800 × *g* for 5 min at 4 °C to remove ADSCs and then at 2,000 × *g* for 10 min at 4 °C to remove cellular debris. The supernatant was the centrifuged at 100,000 × *g* for 2 h at 4 °C in a Beckman Coulter Optima XPN-100 Ultracentrifuge using an SW 41 Ti rotor. The supernatant was discarded and the pellet was washed with 12 mL phosphate-buffered saline (PBS), followed by a second ultracentrifugation at 100,000 × *g* for 2 h at 4 °C. The supernatant was discarded and EVs were resuspended in 100 μL PBS. The biological characteristics of EVs were analyzed by TEM using nanoparticle tracking analysis (NTA; NanoSight LM10), and immunoblotting.

### Loading of siRNAs into EVs

The purified EVs were washed twice in Cytoporation® Media T (BTXpress, USA), and then 50 μg EVs (≈1 × 10^10^ particles) were mixed with 50 μg siRNAs containing a 2′-O-methyl modification on every nucleotide and 3′ phosphorothioate internucleotide linkages at the first three 5′ and 3′ nucleotides (Genepharma, China) in 100 μL Cytoporation® Media T. The mixture was electroporated at 400 V in a 2-mm electroporation cuvette (BTXpress). The EVs were purified from the mixture and unloaded siRNAs were removed using a SuperEV 0.5 purification column (Rengen Biosciences, China).

### Decoration of EVs with an anti-F4/80 antibody

To analyze the targeted delivery of loaded siRNAs-EVs to macrophages in vivo, a rat monoclonal anti-F4/80 antibody (Abcam, USA) was used to decorate the surface of ADSC-derived EVs with an EV decorating kit (Rengen Biosciences), in accordance with the manufacturer’s instructions. The biological characteristics of these EVs were analyzed by TEM, NTA (NanoSight LM10), and immunoblotting.

### Tracking of EVs in vitro and in vivo

To visualize the influence of ADSC-derived EVs on the polarization of macrophages in vitro, EVs were labeled with 2 μM 1,1'-dioctadecyl-3,3,3',3'-tetramethylindocarbocyanine perchlorate (Dil, excitation/emission [Ex/Em] = 549/569 nm; Coolaber, Beijing, China,) for 1 h at 37 °C and then purified with a SuperEV 0.5 purification column. On the basis of a previous report [13], macrophages were incubated with EVs (1 × 10^8^ particles/mL) for 24 or 48 h. The fluorescence intensity of NF-κB1 (p50; FITC, Ex/Em = 494/518 nm) and NF-κB (p65; Cy5, Ex/Em = 560/640 nm) in the nucleus was assessed using a Leica TCS-SP8 confocal microscope and ImageJ. To track ADSC-derived EVs in vivo, EVs were labeled with 2 μM DiR (Absin, Shanghai, China) for 1 h at 37 °C and then purified with a SuperEV 0.5 purification column. F4/80-EVs (1 × 10^9^ particles/mouse) were intrauterine injected into mice. At 24 h after injection, the mice were anesthetized and imaged by an NIR-n Imaging System-H (Series III 900/1700, NIROPTICS, Suzhou, China). To further analyze the location of F4/80-EV compounds in tissues, pancreatic tissue was selected, fixed, sectioned, and immunostained with an anti-rat antibody (FITC-conjugated antibody, Zhongshan Golden Bridge, Beijing, China), rabbit monoclonal anti-F4/80 antibody (Abcam), and anti-rabbit antibody (Cy5-conjugated antibody, Zhongshan Golden Bridge).

### Statistical analysis

All experiments were performed independently at least three times and repeated in triplicate. Results are presented as the mean ± standard error of the mean (SEM). Differences were assessed using Student’s t-test (two groups) or one-way analysis of variance (ANOVA, more than two groups), unless noted otherwise. A p value < 0.05 was considered statistically significant; ns indicates non-statistical significance by Student’s t-test or one-way ANOVA; ∗ p < 0.05, ∗∗ p < 0.01, and ∗∗∗ p < 0.001.

## Supplementary Information

**Additional file 1: Figure S1**.The analysis of biological characteristics in human ADSCs.The ADSCs had successfully differentiated into chondrocytes(A), adipocytes(B), and osteoblasts(C). CD29, CD90, and CD105 were positive in human ADSCs(D). **Figure S2**.The ADSC-secreted EVs were incubated with M1 phenotype macrophages. Fluorescence images were used to evaluate localization of FITC-labeled NF-κB1 (p50) and Cy5-labeled NF-κB(p65). FQ,fluorescence quantitation; a.u., arbitrary unit. **FigureS3**. Internalizationof normal EVs and F4/80-EVs into M1 phenotype macrophages. Microscopic visualizationof internalization(left), and analysisof EVs fluorescence intensity(right). **p< 0.01. **Figure S4**. Flow cytometry of M1 (iNOS) and M2 (CD206) macrophage markers after treatment with various F4/80-EVsand quantification of iNOS and CD206 expression levels in various F4/80-EV-treated M2 phenotype macrophages.ns, not significant. **Figure S5**.Bio-toxicity analysis of the various F4/80-EVsin mice.n=5,ns, not significant, *p< 0.05, **p< 0.01, ***p< 0.001. **Figure S6**. Whole blot images for Figure 4. **Figure S7**. Whole blot images for Figure 5.

## Data Availability

The datasets used and/or analyzed during the current study are available from the corresponding author on reasonable request.

## Abbreviations

PROM: Premature rupture of membranes
ceRNA: Competing endogenous RNAs
TNF: Tumor necrosis factor
MMP: Matrix metalloproteinase
circRNAs: Circular RNAs
miRNAs: MicroRNAs
EVs: Extracellular vesicles
ADSCs: Adipose tissue-derived stem cells
CS: Caesarean section
iNOS: Inducible Nitric Oxide Synthase
PCR: Polymerase Chain Reaction
ELSIA: Enzyme Linked Immunosorbent Assay
cir2047: CircRNA_0002047
AGO2: Argonaute RISC catalytic component 2
NF-κB: Nuclear factor kappa B subunit
FITC: Fluorescein isothiocyanate
Cy5: Sulfo-Cyanine5
Dil: 1,1-Dioctadecyl-3,3,3,3-tetramethylindocarbocyanine perchlorate
DiR: 1,1'-Dioctadecyl-3,3,3',3'-Tetramethylindotricarbocyanine Iodide
