# Supplementary material for: Uterine macrophages as treatment targets for therapy of premature rupture of membranes by modified ADSC-EVs through a circRNA/miRNA/NF-κB pathway
Source: J Nanobiotechnology. 2022 Nov 19;20:487. doi: 10.1186/s12951-022-01696-z (PMC9675163; doi:10.1186/s12951-022-01696-z)
Supplement: Supplementary file 1 — Additional file 1: Figure S1.The analysis of biological characteristics in human ADSCs.The ADSCs had successfully differentiated into chondrocytes(A), adipocytes(B), and osteoblasts(C). CD29, CD90, and CD105 were positive in human ADSCs(D). Figure S2.The ADSC-secreted EVs were incubated with M1 phenotype macrophages. Fluorescence images were used to evaluate localization of FITC-labeled NF-κB1 (p50) and Cy5-labeled NF-κB(p65). FQ,fluorescence quantitation; a.u., arbitrary unit. FigureS3. Internalizationof normal EVs and F4/80-EVs into M1 phenotype macrophages. Microscopic visualizationof internalization(left), and analysisof EVs fluorescence intensity(right). **p< 0.01. Figure S4. Flow cytometry of M1 (iNOS) and M2 (CD206) macrophage markers after treatment with various F4/80-EVsand quantification of iNOS and CD206 expression levels in various F4/80-EV-treated M2 phenotype macrophages.ns, not significant. Figure S5.Bio-toxicity analysis of the various F4/80-EVsin mice.n=5,ns, not significant, *p< 0.05, **p< 0.01, ***p< 0.001. Figure S6. Whole blot images for Figure 4. Figure S7. Whole blot images for Figure 5. [file 12951_2022_1696_MOESM1_ESM.pdf]

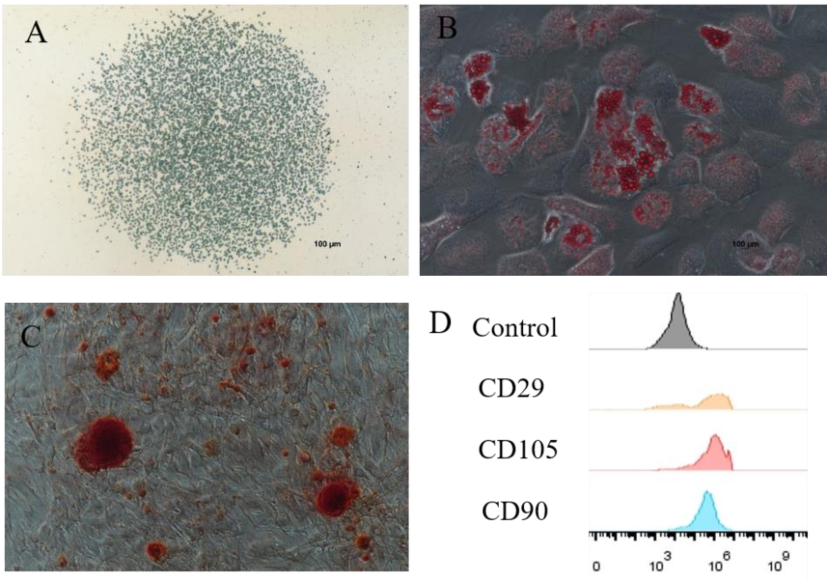

Figure S1. The analysis of biological characteristics in human ADSCs. The ADSCs had successfully differentiated into chondrocytes (A), adipocytes (B), and osteoblasts (C). CD29, CD90, and CD105 were positive in human ADSCs (D).

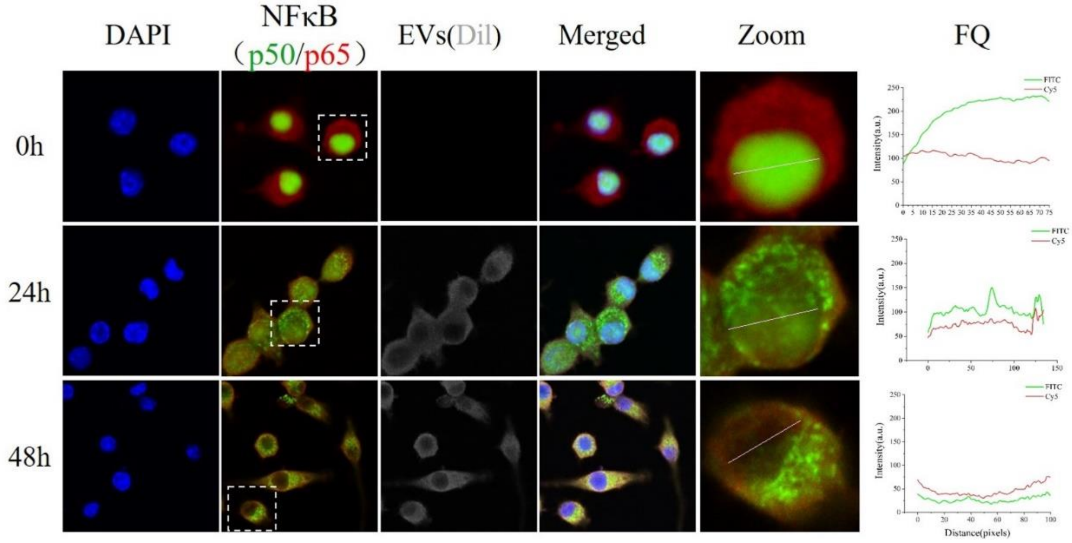

Figure S2. The ADSC-secreted EVs were incubated with M1 phenotype macrophages. Fluorescence images were used to evaluate localization of FITC-labeled NF-κB1 (p50) and Cy5-labeled NF-κB (p65). FQ, fluorescence quantitation; a.u., arbitrary unit.

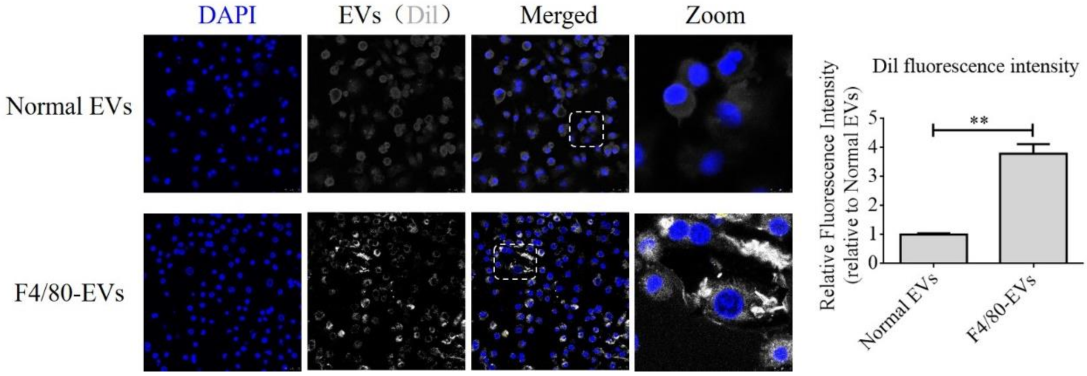

Figure S3. Internalization of normal EVs and F4/80-EVs into M1 phenotype macrophages. Microscopic visualization of internalization (left), and analysis of EVs fluorescence intensity (right). ***p* < 0.01.

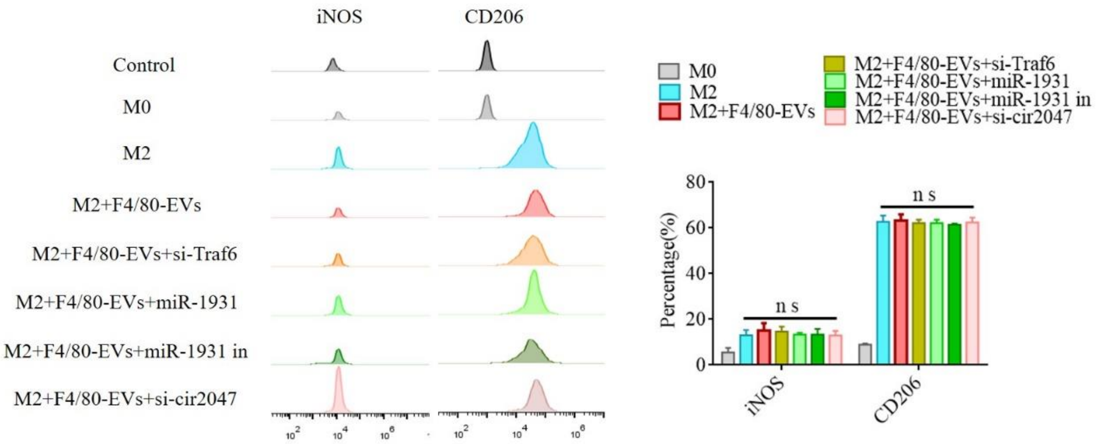

Figure S4. Flow cytometry of M1 (iNOS) and M2 (CD206) macrophage markers after treatment with various F4/80-EVs and quantification of iNOS and CD206 expression levels in various F4/80-EV-treated M2 phenotype macrophages. n s, not significant.

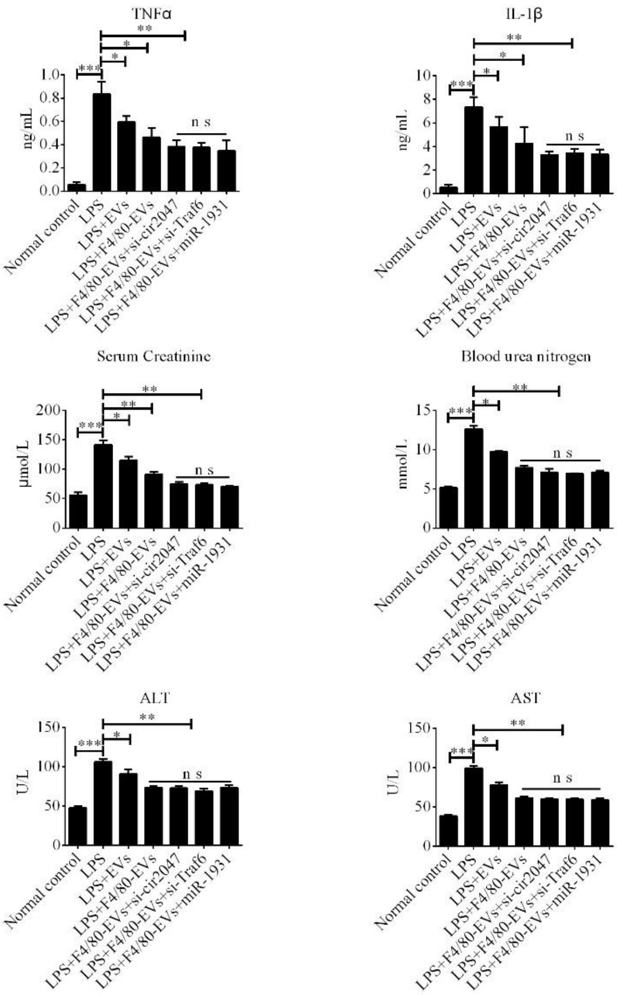

Figure S5. Bio-toxicity analysis of the various F4/80-EVs in mice. n=5, n s, not significant, *$p < 0.05$, **$p < 0.01$, ***$p < 0.001$.

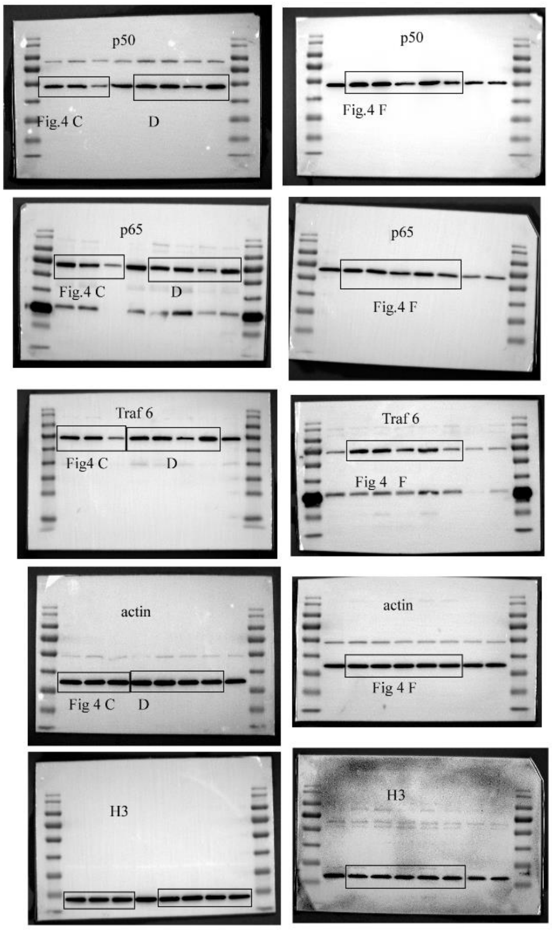

Figure S6. Whole blot images for Figure 4.

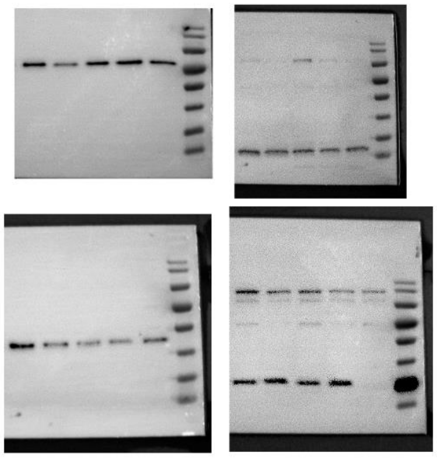

Figure S7. Whole blot images for Figure 5.
